# Surgical treatment was desirable to improve neuromuscular function in patients with sustained 3 years fracture‐dislocation of lower cervical spine: A case report

**DOI:** 10.1002/ibra.12054

**Published:** 2022-07-20

**Authors:** Hao Yuan, Yu Pi, Chong Wang, Jin‐Cheng Si Ma, Sheng Liu, Jun Ao

**Affiliations:** ^1^ Department of Orthopaedic Surgery Affiliated Hospital of Zunyi Medical University Zunyi Guizhou China; ^2^ Department of Anesthesiology Southwest Medical University Luzhou Sichuan China; ^3^ Pharmacology Institute Heidelberg University Heidelberg Germany

**Keywords:** internal fixation, lower cervical spine, old fracture dislocation, neural restoration, neurosurgery, titanium mesh cage

## Abstract

To investigate the changes in neuromuscular function of anterior approach combined with subtotal vertebral body resection and titanium mesh cage (TMC) internal fixation for the old fracture‐dislocated lower cervical spine. A 56‐year‐old female was admitted to the hospital with neck pain and numbness of the left upper extremity for 3 years due to a fall injury from a height, which worsened for 20 days. Although 3 years had passed, the patient still had significant left limb numbness and decreased muscle strength. Eventually, the patient was diagnosed with the old fracture‐dislocation type injury of C6 and C7. C6 was II‐degree anterior dislocation and the bilateral joint process was locked, C7 was burst fracture, and C5 was spinal cord segment injury. Then, the operation of the anterior approach combined with subtotal vertebral body resection and TMC internal fixation was performed under general anesthesia. Postoperative symptoms were significantly improved. And during five‐year of follow‐up, no adverse reactions and complications were reported. Although cervical fracture and dislocation combined with cervical spinal cord injury had persisted for many years, surgical treatment was necessary. The anterior approach combined with subtotal vertebral body resection and TMC internal fixation was desirable to improve neuromuscular function for the old fracture‐dislocation of the lower cervical spine, which has some guiding effects on the clinical treatment.

## Graphical abstract

Old fracture‐dislocations of the lower cervical spine are common and are associated with varying degrees of spinal cord and nerve root injury. The purpose of this study was to investigate the changes in neuromuscular function of the anterior approach combined with subtotal vertebral body resection and titanium mesh cage (TMC) internal fixation for the old fracture‐dislocated lower cervical spine. This study showed that although cervical fracture and dislocation combined with cervical spinal cord injury had persisted for many years, surgical treatment was necessary. The anterior approach combined with subtotal vertebral body resection and TMC internal fixation was desirable to improve neuromuscular function for the old fracture‐dislocation of the lower cervical spine, which has some guiding effects on clinical treatment.

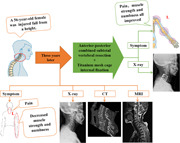

## 1 INTRODUCTION

The early diagnosis and treatment of fracture‐dislocation of the cervical vertebra can improve recovery and prevent further deterioration. Fracture‐dislocation of the cervical vertebra is considered old dislocation if it lasts for more than 3 weeks and remains untreated. The old fracture‐dislocation of the lower cervical spine was more common, most of which were accompanied by varying degrees of the spinal cord and nerve root injury. And due to the continuous dislocation and the obvious instability of the injured segment, the spinal cord injury was more aggravated.^1^ The treatment principle was complete decompression, reconstruction of the cervical spine stability, and restoring the physiological curve of the cervical spine as much as possible.^2^ The nerve function of the injured segment has been partially recovered and was in one‐stage due to the regulation of the patient with old fracture‐dislocation of cervical spine trauma. The clinical efficacy of the anterior cervical subtotal vertebral body resection or titanium mesh cage (TMC) internal fixation in the treatment of vertebral injury has been supported by some pieces of literature.^3^, ^4^, ^5^ However, there were few cases of the anterior approach combined with subtotal vertebral body resection and TMC internal fixation for partial recovery of old fracture‐dislocation of cervical spine trauma. In addition, the majority of old cervical fractures are operated on within months of injury, and patients requiring surgical treatment several years after injury were rarely reported. So, in this case, we report the treatment outcome after the anterior approach combined with subtotal vertebral body resection and TMC internal fixation in a female patient who suffered fracture‐dislocation of the inferior cervical spine for 3 years.

## 2 CASE REPORT

**Figure 1:**
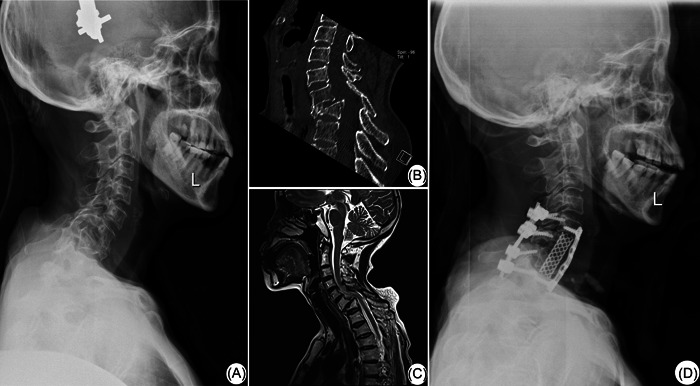
Preoperative and postoperative imaging examinations of the patient. A 56‐year‐old female was admitted to the hospital with neck pain and numbness of the left upper extremity for 3 years due to a fall injury from a height, which worsened for 20 days. Preoperative X‐ray radiography (A), CT (B), and MRI (C) indicated the dislocation of the C6 and C7, burst fracture of the C7, and posterior protrusion. Postoperative X‐ray radiography (D) showed that C6 and C7 subtotal vertebrae resection with TMC and internal fixation were in a good position. C, cervical vertebra; CT, computed tomography; MRI, magnetic resonance imaging.

A 56‐year‐old female was admitted to the hospital with neck pain and numbness of the left upper extremity for 3 years due to a fall injury from a height, which worsened for 20 days. The patient was in a passive position with restricted head and neck movement. Pain occurs when the patient moves the neck. The patient experienced more severe pain by pressing down on the cervical spine and the spinal canal. The muscle strength examination of limbs suggested that the muscle strength of the left limb decreased significantly, accompanied by numbness and discomfort (Table 1). The abdominal wall reflex, perianal reflex, Achilles tendon reflex, and knee‐tendon reflex was not weakened. The results of Hoffman's sign, Babinski's sign, patella clonus, and ankle clonus were negative. The nerve function of the injured segment was partially recovered, but only reached one‐stage, and there were still different degrees of the spinal cord or nerve root damage. The imaging findings suggested dislocation of Cervical (C) 6 and C7, burst fracture of C7, and burst into the spinal canal to compress the spinal cord (Figure 1A–C). According to the history of trauma, physical examination, and preoperative examination, the patient was diagnosed with an old fracture‐dislocation type injury of C6 and C7. C6 was II‐degree anterior dislocation and the bilateral joint process was locked, C7 was burst fracture, and C5 was spinal cord segment injury (American Spinal Injury Association [ASIA] C level).

**Table 1:** Preoperative and postoperative muscle strength level tests

Items	Pre	Post
Right shoulder extension, internal collection, flexion, and extension muscles strength	4	4
Right elbow flexion and extension muscles strength	4	4
Right wrist finger flexion and extension muscles strength	4	4
Right lower limb muscle strength	4	4
Left shoulder extension, internal collection, flexion, and extension muscles strength	2	4
Left elbow flexion and extension muscles strength	2	4
Left wrist finger flexion and extension muscles strength	1	3
Left lower limb muscle strength	3	4

Combined with the patient's medical history, to remove the factors causing spinal cord and nerve root compression, completely or partially correct the deformities, restore the normal sequence of the cervical spine and stabilize the cervical spine of corresponding segments, it was proposed to conduct anterior approach combined with subtotal vertebral resection and TMC internal fixation. After admission, the patient was given general treatment such as absolute bed rest, skull traction, and so on. Then, the patients underwent the anterior approach combined with subtotal vertebral resection and TMC internal fixation under general anesthesia and tracheal intubation (Figure 1D). After the operation, the patient was treated with supine position nursing and antibiotics.

At postoperative follow‐up of 3 months, the patient showed that the activity, muscle strength, and sensation of limbs were significantly improved than before the operation. Postoperative physical examination of the patient showed that numbness and muscle strength of the left upper arm was significantly improved than pre‐operation (Table 1). There was no postoperative discomfort and complications. The image showed the subtotal vertebral resection of C6 and C7, and the great fixation position of TMC internal. During three‐year of follow‐up, there were no adverse reactions and complications reported.

## 3 DISCUSSION

This case reports a female patient suffering old fracture‐dislocation of lower cervical spine trauma that lasted for 3 years, whose neurological and spinal functional impairment was significantly improved by anterior approach combined with subtotal vertebral resection and TMC internal fixation. This case further proved that the anterior approach combined with subtotal vertebral resection and TMC internal fixation was effective, safe, and feasible for the treatment of old lower cervical fracture‐dislocation. In addition, this case showed that the timing of surgery for an old cervical fracture was not fixed and may not be limited to a few months after the injury. Surgical treatment was still necessary and effective in patients with old fracture‐dislocation of lower cervical spine trauma lasting for years. The patient recovered well after 5 years of continuous follow‐up after surgery.

There was still a dispute about the time of operation for patients with cervical fracture‐dislocation combined with cervical spinal cord injury. Should you operate immediately after the trauma, or wait for the patient's general condition to stabilize and the spinal edema to subside? The results of animal experiments showed that the recovery of nerve function was inversely proportional to the compression time of the spinal cord.^6^ However, the clinical research results of different periods of operations on spinal cord nerve function recovery were inconsistent.^7^, ^8^ The delay of operation will increase the possibility of aggravating spinal cord injury. It would be safer to wait until the patient stabilizes and the spinal edema subsides.^8^ Some studies showed the factors that determined the recovery of spinal cord function also included the impact of the original injury and the duration of continuous compression.^9^ The operation timing of specific cases must be determined according to the specific pathological characteristics. For the old fracture‐dislocation of the lower cervical spine, the operation should be performed early as soon as found. When cervical spine fracture‐dislocation combined with spinal cord injury, early reduction or removal of bone or disc tissue was beneficial to restore the function of the spinal cord and spinal nerve root.

Patients with old fracture‐dislocation of cervical spine trauma may have delayed the cervical spine surgery due to limited medical conditions in the injured area or injuries in other parts of the body. The time of delay was not a few days or a week after the injury, but more than months. So, the nerve function of the injured segment was partially recovered and only reached one‐stage, and there were still different degrees of the spinal cord or spinal nerve root damage. However, simple skull traction was difficult to cure for most of the old lower cervical spine fracture‐dislocation, and it was often necessary to recover the dislocation vertebral body by operation.^10^ The operation methods of the patient should take into the aspects of decompression of the spinal cord, deformity correction, and reconstruction of cervical stability. The surgical treatment of unstable traumatic injury of the cervical spine can be performed through a posterior approach or anterior approach, each of which has advantages and disadvantages. The anterior approach can directly remove the bone or disc tissue that oppressed the spinal cord or nerve root, and can also implant the bone stably, as well partially correct the deformity and restore the cervical spine sequence. On the one hand, it can restore the function of nerve tissue that is temporarily lost or incomplete due to compression, while on the other, it can prevent spinal cord disease caused by compression or unstable stimulation. So, the anterior approach decompression is considered to be a better decompression method at present.^8^, ^11^, ^12^ Meanwhile, TMC was the common equipment used for interbody fusion in anterior cervical resection and fusion (ACCF), and the fixable TMC can provide enough effective stability reconstruction after the anterior subtotal cervical vertebra resection.^13^ Although the overall fixation strength was not as good as that of traditional TMC and plate fixation mode, it may be more beneficial to the fusion of intervertebral bone graft particles.^14^ The anterior decompression combined with internal fixation can restore cervical curvature, nice reduction effect, complete decompression, and immediate stability after operation for severe fracture‐dislocation of the lower cervical spine.^15^ This literature was consistent with the therapeutic effect of this case, which achieved the full decompression of spinal nerve root, made the dislocation of the cervical spine be restored as much as possible, recovered or partially restored the physiological curve of the cervical spine, and stabilized the pathological segment by the way of intervertebral bone grafting fusion and internal fixation.

One of the factors hindering the recovery of spinal cord function is the compression of bone tissue and disc tissue either caused by anterior dislocation or kyphosis. Surgical treatment can achieve varying degrees of improvement in neurological function regardless of the severity of nerve injury. Delayed anterior decompression and fusion of the cervical spine was still an effective treatment for cervical spinal cord injury caused by cervical fracture‐dislocation. Due to the injured conduction tract of the spinal cord, the remaining active nerve tissue in the patients with complete spinal cord injury can be able to partial recovery of the nerve function of the upper limb, rather than the lower limb. The operation effect in patients with remaining partial sensation at the distal end but complete loss of motor function was similar to those with complete spinal cord injury. Miao et al. found that the nerve root function of patients with complete or incomplete spinal cord injury can be improved by anterior prior partial corpectomy and TMC fusion and internal fixation.^16^ Anderson et al. summarized 51 cases of complete motor paralysis with more than 1 month delay, 25 of them with nerve root function improvement.^17^ Some literature has reported that spinal cord injury can be transformed from completeness to incompleteness after surgery.^18^ Bohlman et al. observed a group of patients with old spinal cord incomplete injury and found that 47 patients of 58 patients had functional improvement after anterior decompression fusion.^19^ Mirza et al. thought that it was difficult to accurately distinguish between complete and incomplete injury in the early stage of spinal cord injury, so it was better to treat patients according to incomplete injury mode.^8^ From the experience of this case, this principle can also be used in the treatment of patients with old cervical fracture‐dislocation and cervical spinal cord injury. The compression or the stimulation of local instability caused by old fracture‐dislocation was one of the obstacles to the further recovery of spinal cord function. In spite of the delay in removing the compression or stimulation factors, it can still make some patients recover spinal cord function to different degrees.

## 4 CLINICAL APPLICATION AND INNOVATION

This case was unique, here the patient had a history of cervical vertebra fractures for several years with poor cervical malunion and incomplete neurological recovery, and the need for surgery was controversial. This was a relatively rare case. The report of the postoperative outcome of this patient was helpful to provide clinical treatment advice for patients with years of old cervical vertebra fractures. Anterior combined subtotal laminectomy has been widely used in clinics and achieved satisfactory results.^20^, ^21^, ^22^ Due to the high incidence of anterior spinal cord compression, this kind of surgery can improve blood flow circulation and prevent secondary pathophysiological processes. TMC has good biocompatibility and can be trimmed in size according to the length of the vertebral space, which facilitates embedding between the vertebrae and prevents them from moving, and also plays a supporting role in the longitudinal direction.^23^, ^24^, ^25^, ^26^ Therefore, anterior decompression combined with subtotal laminectomy combined with TMC internal fixation combines the advantages of both, ensuring relieving spinal cord compression, and promoting bone graft fusion, which is a more ideal surgical approach and worthy of clinical promotion.^27^, ^28^

## 5 LIMITATIONS

Only one patient was reported in this case. So, the reliability of the therapeutic effect was limited. More case studies are needed to determine the effectiveness and necessity of surgical treatment for patients with old cervical fractures.

## 6 CONCLUSIONS

The need for surgical treatment in patients with cervical fracture and dislocation combined with cervical spinal cord injury that has persisted for years is debatable. In this case, the patient with old fracture‐dislocation of the lower cervical spine for 3 years was treated by anterior decompression, partial vertebral resection, and TMC implantation and internal fixation. The symptoms improved significantly after the operation. It is suggested that although the damage has lasted for years, the therapeutic effect of the anterior approach combined with subtotal vertebral resection and TMC internal fixation was effective for old fracture‐dislocation of the lower cervical spine, which can be used in clinical practice.

## AUTHOR CONTRIBUTIONS

Hao Yuan, Chong Wang, and Jin‐Cheng Si Ma performed the operations and collected the clinical data. Yu Pi completed the manuscript. Jun Ao and Sheng Liu gave the overall guidance and participated in the operations and revision of the paper. All authors have read and approved the final submitted manuscript.

## CONFLICT OF INTEREST

The authors declare no conflict of interest.

## ETHICS STATEMENT

The research was conducted ethically in accordance with the Helsinki Declaration and approved by the Biomedical Research Ethics Committee of the Affiliated Hospital of Zunyi Medical University (No. KLL‐2020‐275). The patient provided written consent to publish this case, including the use of images. [Correction added on 7 December 2023 after first online publication: This section was revised at the request of authors.]

## Data Availability

The datasets used and/or analyzed during the current study are available from the corresponding author on reasonable request.
